# Supramolecular Deep Eutectic Solvents as a Janus Green Platform: Integrating Curcuminoid Extraction and Biopolymer

**DOI:** 10.3390/molecules31122104

**Published:** 2026-06-15

**Authors:** Clelia Aimone, Giorgio Capaldi, Emanuela Calcio Gaudino, Anastasia Anceschi, Alessia Patrucco, Kristina Radošević, Giorgio Grillo, Giancarlo Cravotto

**Affiliations:** 1 Department of Drug Science and Technology, University of Turin, Via P. Giuria 9, 10125 Turin, Italy; clelia.aimone@unito.it (C.A.); giorgio.capaldi@unito.it (G.C.); emanuela.calcio@unito.it (E.C.G.); giancarlo.cravotto@unito.it (G.C.); 2 Institute of Intelligent Industrial Technologies and Systems for Advanced Manufacturing (STIIMA), Italian National Research Council (CNR), Corso G. Pella 16, 13900 Biella, Italy; anastasia.anceschi@stiima.cnr.it (A.A.); alessia.patrucco@stiima.cnr.it (A.P.); 3 Laboratory for Cell Cultures, Applications and Biotransformations, Department of Biochemical Engineering, Faculty of Food Technology and Biotechnology, University of Zagreb, Pierottojeva Ulica 6, 10000 Zagreb, Croatia

**Keywords:** deep eutectic solvents, SupraDES, curcuminoids, biopolymers, cyclodextrins, green extraction, stability, BHMF

## Abstract

Curcuminoids from *Curcuma longa* L. (curcumin, demethoxycurcumin, bisdemethoxycurcumin) are attractive bioactives yet constrained by low water solubility and chemical instability. Herein, we introduce a Supramolecular Deep Eutectic Solvent (SupraDES) as a “Janus” green platform, combining extraction and stabilization with a subsequent solvent-to-material strategy. Eight NaDES/SupraDES formulations based on choline chloride (ChCl) or betaine with glycerol (Gly) or citric acid (CitA), with/without β-cyclodextrin (βCD), were assessed. The extinction coefficients of the most promising solvents were extrapolated at 425 nm for the UV–vis quantification of curcuminoids, to determine extraction performance. The SupraDES ChCl:Gly:βCD gave the best performance during the first solvent screening, improving at the same time the bioactive stability (after 30-day, 47.5% loss vs. 62.8% of ChCl:Gly alone). Subsequent microwave-assisted extraction (MAE) optimization identified 80 °C as the optimal process temperature, with near-equilibrium reached within 15 min (3139.4 µgCurc/gEXT). Peleg modelling (R^2^ = 0.997) indicated a fast extraction rate and limited benefit from longer residence times. Finally, the curcuminoid-loaded SupraDES was incorporated into polyvinyl alcohol (PVA) networks crosslinked with CitA and 2,5-bis(hydroxymethyl)furan (BHMF)*;* thermal analysis confirmed the formation of a stable crosslinked structure. To the best of our knowledge, this is the first report of a βCD-based SupraDES acting as a Janus platform that couples supramolecular extraction of lipophilic bioactives with their direct incorporation into bio-based polymeric materials, exemplifying an integrated green chemistry approach aligned with circular bioeconomy principles.

## 1. Introduction

Curcuminoids (primarily curcumin, demethoxycurcumin, and bisdemethoxycurcumin), are the main bioactive polyphenols found in the rhizomes of *Curcuma longa* L., commonly known as turmeric [1]. These compounds have garnered substantial industrial and academic interest due to their wide-ranging biological properties, including potent antioxidant, anti-inflammatory, anticancer, antimicrobial, neuroprotective, and cardioprotective activities [2]. However, their broader application in food, pharmaceutical, and biomedical sectors is significantly limited by their low water solubility and chemical instability under light, heat, and varying pH conditions [3,4,5].

Conventional extraction methods, such as Soxhlet, maceration, or solvent extraction, are often inefficient, time-consuming, and reliant on hazardous organic solvents, which may degrade curcuminoids and produce environmentally harmful waste. In response, the development of green extraction technologies has gained momentum, with techniques such as microwave-assisted extraction (MAE), ultrasound-assisted extraction and supercritical fluid extraction offering improved efficiency and sustainability [6,7,8].

Among these innovations, Natural Deep Eutectic Solvents (NaDES) have emerged as a promising green alternative. They are formed from combinations of natural hydrogen bond donors (HBDs) and acceptors (HBAs), such as ammonium salts, organic acids, sugars and polyols [9,10]. NaDES exhibit several key advantages: biodegradability, biocompatibility, tunable polarity, and an exceptional ability to solubilize both hydrophilic and hydrophobic bioactives (such as curcuminoids) [11]. Moreover, their low volatility and non-toxic nature make them ideal for sustainable applications in extraction and formulation [12,13,14,15].

Recent advances have introduced a powerful subclass known as Supramolecular Deep Eutectic Solvents (SupraDES), which integrate host molecules (notably cyclodextrins) into the DES matrix [16]. These systems leverage host–guest molecular recognition to enhance extraction efficiency, chemical stability and the functionality of the recovered compounds [17,18,19]. As Zhou et al. (2024) [20] highlight, SupraDES systems offer multifunctionality through rational molecular design, enabling them to act both as solvent media and functional components in downstream applications. Unlike conventional NaDES composed of small hydrogen-bonding components, the proposed SupraDES incorporates the β-cyclodextrin (βCD) as a cyclic oligostructural host within a hydrophilic DES matrix (based on choline chloride or betaine combined with glycerol or citric acid). The amphiphilic architecture of βCD, hydrophilic exterior and lipophilic cavity enables host–guest inclusion of curcuminoids, thus allowing efficient extraction of lipophilic compounds in a hydrophilic medium while simultaneously enhancing their stability.

The “Janus” nature of NaDES and SupraDES lies in their simultaneous dualistic ability to:

1. Serve as highly effective extraction media, improving metabolites’ solubilization and chemical stability through hydrogen bonding or supramolecular interactions;
2. Function as reactive matrices or structural precursors in the development of bio-based polymer systems.

This integrated approach exemplifies green chemistry principles and aligns with circular bioeconomy goals by minimizing synthetic additives, enabling solvent reuse, and embedding bioactivity within biodegradable polymeric networks [21,22].

The present study exploits the dual-functionality Janus feature of NaDES and SupraDES, particularly those based on choline chloride (ChCl), betaine (Bet), citric acid (CitA), glycerol (Gly), and βCD, for the green extraction of curcuminoids from *Curcuma longa* L. The resulting extracts will subsequently be employed for the fabrication of polymeric materials based on polyvinyl alcohol (PVA) and natural pectin, using 2,5-bis(hydroxymethyl)furan (BHMF) as a bio-derived crosslinker. Natural pectin is widely used due to its distinctive physicochemical properties, particularly its ability to act as gelling, thickening, and stabilizing agents in various applications, along with its bioactivity [23,24]. It can be extracted using various approaches, from traditional methods such as acid extraction or enzymatic treatments to more innovative extraction technologies [25,26,27,28].

The adopted strategy transforms the DES medium from a solvent phase into a functional component of the polymer matrix, forming a bio-based material system with embedded bioactivity and structural integrity, exemplifying the Janus dual behaviour in supramolecular solvent systems. To the best of our knowledge, this represents the first report of a β-CD-based SupraDES system enabling both the extraction of curcuminoids and their direct incorporation into a biopolymer matrix within a single integrated process.

This work addresses three interconnected aims: (i) to evaluate the efficacy of various NaDES and SupraDES systems in extracting curcuminoids from *Curcuma longa* L.; (ii) to investigate the supramolecular stabilization and solubilization mechanisms involved in curcuminoid–NaDES interactions; and (iii) to demonstrate the feasibility of using these DES systems as structural and functional precursors in the formation of green polymeric materials.

## 2. Results and Discussion

### 2.1. NaDES/SupraDES-Assisted Curcuminoid Extraction

#### 2.1.1. Component Selection

As stated in the introduction, the eutectic systems were selected focusing the attention on the two “Janus” roles of the solvent: effective extraction and serving as a reactive matrix for biopolymer production. In this context, the initial selection focused on the ChCl:Gly system, based on evidence reported in the existing literature [29]. The second option identified was a citric acid-based DES, specifically when formulated in combination with ChCl [30]. Taking the lead from those premises, ChCl:Gly and ChCl:CitA were adopted as references with two screened modification: (i) the substitution of ChCl with Bet and (ii) the addition of βCD to create a SupraDES.

Bet was investigated as a replacement for ChCl due to its applicability in food applications; conversely, the latter potentially opens new scenarios for the biopolymer’s use [31]. On the contrary, the interest toward the SupraDES, raised from the double feature of βCD to enhance curcuminoid stability and to act as a reactive precursor to synthesize polymeric matrixes (in particular with CitA) [30].

Different tests were conducted to identify the best molar ratio in terms of stability and exploitability, for the following systems: ChCl:Gly, ChCl:Gly:βCD, Bet:Gly, Bet:Gly:βCD, ChCl:CitA, ChCl:CitA:βCD, Bet:CitA, Bet:CitA:βCD. To help the eutectic’s formation, minimal amount of water was evaluated as well. The results are summarized in Table 1.

It is worth noting that across the series, CitA-based systems require higher formation temperatures than Gly-based ones: both ChCl:CitA and Bet:CitA form at 100 °C, whereas Gly-containing systems form at 60–80 °C. βCD generally decreases the formation temperature. In contrast, Bet:Gly already forms at 60 °C, so no lower formation tests were made on Bet:Gly:βCD. Cyclodextin incorporation appears to facilitate SupraDES formation under milder thermal conditions, consistent with its participation in the supramolecular H-bond network and with reports of melting-point depression in βCD-based eutectic systems [32].

**Figure 1:**
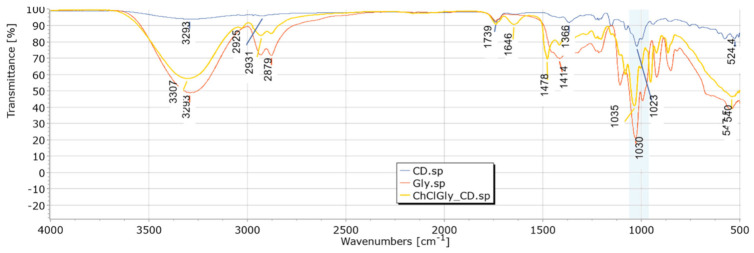
ATR-FTIR spectrum of SupraDES (ChClGly_CD; yellow) and its forming component: cyclodextrin (CD; blue) and glycerol (Gly; orange). Light blue area points out the diagnostic peaks.

To verify that the addition of βCD resulted in the formation of a supramolecular network within the DES rather than a simple physical dispersion of CD within the DES, ATR-FTIR analysis was performed (see Figure 1). It is noteworthy that the preparation of SupraDES is a well-known and documented phenomenon [16]. Moreover, as already stated, the observation of a moderate depression in the melting point (20 °C lower than that of their corresponding NaDES counterpart) provides significant evidence. This thermal behaviour suggests that the addition of CD to the mixture of the forming DES led to a formation of a more complex architecture characterized by an extensive network of hydrogen bonds.

The formation of the SupraDES molecular network was further demonstrated by ATR-FTIR analysis. As depicted in the spectra below, a blueshift of the C-O signals (highlighted region in the spectrum) of the formed mixture with respect to the starting components corroborates the hypothesis of formation of the SupraDES.

As mentioned in the Introduction, the formation of several SupraDES, based on various types of CD, have already been widely reported in the literature. Thus, relying on previous studies, it has been possible to properly translate ATR-FTIR spectra. By analyzing the profile of the SupraDES (ChCl:Gly:CD) in comparison with the profiles of CD and Gly, it is possible to observe that all three samples exhibit signals in the fingerprint C–O stretching region (approximately 1020–1040 cm^−1^). Notably, the SupraDES displays a clear blueshift compared to both starting materials, moving from 1023 cm^−1^ (pure βCD) and 1030 cm^−1^ (pure Glycerol) to 1040 cm^−1^ (ChClGlyCD). These blueshifts indicate an increase in the bond force constant of the C–O stretching vibrations after SupraDES formation. This phenomenon suggests that the dense hydrogen-bonding network and the steric confinement within the eutectic matrix induce a conformational restriction and a shortening of the covalent bonds. This confirms that the supramolecular interactions are significantly altering the molecular energy environment of both Gly and CD, leading to a more rigid and stable system. These results are in agreement with literature references, where the formation of a SupraDES based on CD and levulinic acid led to a blueshift of 5–9 cm^−1^ in the C–O stretching region [33]. In our system, the observed blueshift is slightly more pronounced, reaching values of up to ($\Delta v=\;5\text{–}12\;{cm}^{-1}$). This suggests a significant modification in the molecular interactions, likely due to the involvement of three components (ChCl, Glycerol, and CD) which increases the probability and density of hydrogen bonds. The ChCl:Gly matrix appears to be more effective in establishing these supramolecular interactions compared to simpler systems, possibly due to the high number of hydroxyl groups provided by glycerol and the stabilizing presence of the choline cation. Future studies would exploit 2D NMR characterizations (e.g., NOESY) to further elucidate the specific interactions between DES components and their molecular structures.

#### 2.1.2. NaDES Screening and UV-Vis Calibration

The NaDES/SupraDES systems produced have been firstly investigated for the recovery of curcuminoids from *Curcuma longa* L. rhizome’s powder. To perform a preliminary screening, minimize the risk of degradation and ideally isolate as much as possible the contribution of the solvent/metabolite affinity. The first set of extractions were performed at RT, protected from the light. Results helped to exclude Bet:CitA and Bet:CitA:βCD as strong degradation occurred during the procedure, with visible colour change and precipitate formation even after the biomass separation. This outcome is in line with low-pH curcuminoids’ instability and it was possible to state that βCD does not exert any protective role. Thus, after Section 2.1.1 and NaDES Screening, the CitA-based DES have been excluded from the study, even if they have the most promise in terms of polymerizability, according to the literature [30].

To determine the curcuminoid content in the remaining samples, the UV-Vis quantification was adopted as an easy and fast protocol, based on its strong foundation in the literature. This approach is commonly used on curcuminoids due to their high chromophoric power, solvatochromism, and the characteristic absorption wavelength (425 nm) [30].

The UV-Vis quantification protocol relies on Lambert–Beer law and is proportional to ε, an intrinsic intensive property of the analyte, at fixed nm. Different extinction coefficient (ε) values for curcuminoids can be tabulated for different common solvents, such as EtOH (5.5 × 10^4^ L·mol^−1^·cm^−1^) or acetone (4.0 × 10^4^ L·mol^−1^·cm^−1^). Less ordinary solvents are harder to be found and need to be extrapolated by suitable calibration curves, built with standard sequential dilution. For that reason, Gly-based DES have been investigated accordingly, determining ε at 425 nm, as reported in Table 2, together with the related fitting (see also Appendix A, Figure A2A,B).

**Figure 2:**
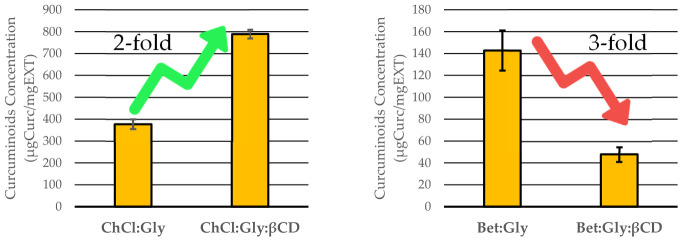
Gly-based NaDES/SupraDES extraction yield of curcuminoids: preliminary screening (RT, 2 h).

Exploiting the calculated values, it was possible to determine the curcuminoid yields for the preliminary extractions performed at RT, hence providing an efficiency scale for the different systems. Figure 2 shows the extraction trend for the four investigated samples, pointing out two different trends. Firstly, Bet-based solvents are less efficient, with a negative contribution of βCD, which decreases the performance by almost 3-fold (142.72 µgCurc/gDES vs. 47.83 µgCurc/gDES, respectively). On the other hand, the Gly-based DES are the best-performing ones, supporting previous literature findings. An interesting point is that in this case the SuprDES form strongly, increasing the extraction features, achieving 788.82 µgCurc/gDES, with a 2-fold increase, in comparison to ChCl:Gly (377.90 µgCurc/gDES). The ChCl:Gly:βCD system, as a ternary SupraDES, can be rationally framed as a hybrid platform that merges two well-established literature features: (i) ChCl:Gly NaDES are among the most effective media for turmeric curcuminoid extraction, (ii) βCD form inclusion complexes with curcuminoids, improving their handling and stability. The fact that ChCl:Gly:βCD delivers *approx*. 2 times higher curcuminoid yield than the βCD-free NaDES indicates that cyclodextrins are contributing functionally to the extraction mechanism: beyond the intrinsic solvating power of the Gly-based system, βCD likely introduces an additional host–guest capture/solubilization pathway that keeps curcuminoids effectively bound and soluble within the SupraDES microenvironment, thereby sustaining a higher driving force for mass transfer. In practical terms, the SupraDES acts simultaneously as an enhanced extraction matrix and a supramolecular reservoir, which is fully consistent with the improved robustness observed.

To the best of our knowledge, this is the first study to apply a SupraDES-based synergistic approach specifically to curcuminoids throughout the entire process, from extraction to a stabilized product. In the next paragraph the latter topic has been investigated beyond the extraction stage, in the context of shelf-life extension.

**Figure 3:**
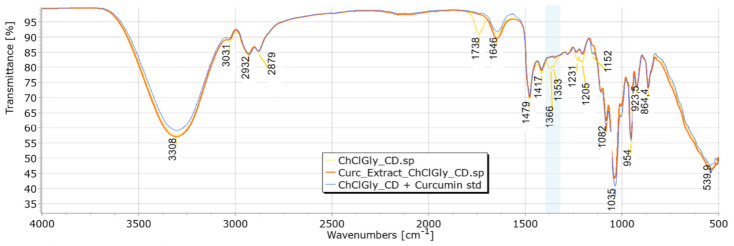
ATR-FTIR analysis to investigate the formation of inclusion complex: spectrum of blank SupraDES (ChClGlyCD; yellow), curcuminoid extract ChClGlyCD-based (Curc_Extract_ChGly_CD; orange) and a formed mixture of SupraDES and curcumin standard (ChGly_CD + Curcumin std; blue). Light blue area points out the diagnostic peaks.

To further investigate the interaction between CD involved in the SupraDES network and curcuminoids extracted by the system, ATR-FTIR was carried out. Spectra are present in Figure 3.

The formation of inclusion complexes between curcumin and β-cyclodextrin is a deeply investigated phenomenon, demonstrated and exploited in various applications. Among those, shelf-life extension has been observed as well. With these premises, in relation to the curcuminoids’ stability enhancement in the presence of SupraDES (See Section 2.1.3), FTIR investigation was conducted to evaluate the role of βCD and the formation of the inclusion complex in the deep eutectic environment.

A comparison between the ATR-FTIR spectra of the blank SupraDES (ChCl:Gly:CD) and the *Curcuma longa* L. extract provides evidence of inclusion complex formation. In the fingerprint region, the signals at 1366–1353 cm^−1^, attributable to the C–H [34] and O–H bending vibrations of the glucopyranose units [35,36], show a significant decrease in intensity. This attenuation suggests that the formation of the inclusion complex restricts the vibrational freedom of these groups, likely due to the establishment of a hydrogen-bonding network between the curcumin and the cyclodextrin host. To further prove the concept an experiment was carried out by mixing the SupraDES with standard curcumin, to evaluate the possible interference with other molecules of the extract or eventual inactivation of βCD during the extraction protocol. Spectra of extract and SupraDES + standard curcumin are completely overlapped. The signal at 1645 cm^−1^ remains a constant signature of water bending within the hydrated SupraDES network.

Furthermore, the characteristic absorption bands of pure curcumin [37,38] are notably absent in the extract spectrum. Since curcumin is highly lipophilic and poorly soluble in the polar DES environment, its spectral absence of signals hinders its successful encapsulation within the apolar and hydrophobic internal cavity of the CD. This masking effect is a well-documented phenomenon in the literature for host–guest systems, indicating that the guest molecule is no longer in a free state but is molecularly dispersed within the host structure, typically between two molecules of CD.

#### 2.1.3. Shelf-Life Evaluation

**Figure 4:**
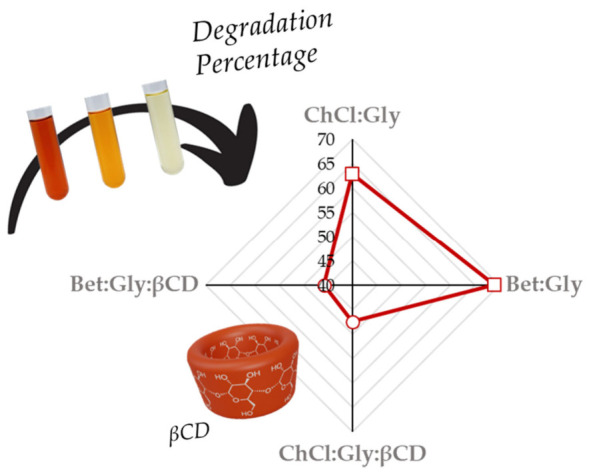
Degradation percentage, expressed as curcuminoid loss after 30 days of storage at RT. Values are normalized on the starting value of each sample.

Curcuminoids are known to benefit from stabilization by NaDES (through strong solvation and low water activity) as well as by cyclodextrins alone (via inclusion complexation that improves solubility and reduces degradation/precipitation). Building on our extraction results, the next step was to investigate the effective stabilization properties of the different systems, either when provided with βCD or not. Figure 4 reports the results of the degradation percentage of each sample, after 30 days of storage at RT and when protected from light (values normalized to starting concentration).

When observing the collected data, it is possible to notice that the two binary systems, independently of their initial curcuminoid concentration, possess degradation values above 60%, with Bet as the most instable solvent. Similarly, the addition of βCD in both cases improves curcuminoid stability, thus supporting shelf-life extension beyond the NaDES framework alone.

When analyzing the two families of components, the following occurs.

- *ChCl-based DES*: degradation drops from 62.77% to 47.52% (−15.25 points, 1.3-fold reduction).
- *Bet-based DES*: degradation drops from 69.01% to 45.81% (−23.20 points, 1.5-fold reduction).

Notably, βCD addition not only reduces degradation but also overcomes the poor baseline stability of the Bet system, making Bet:Gly:βCD the most stabilized formulation. This can support the hypothesis that SupraDES can act as a supramolecular reservoir by the host–guest sequestration, limiting reactive/free curcuminoids during storage.

When the extraction efficiency (Section 2.1.2) and 30-day shelf-life (Section 2.1.3) are considered together, ChCl:Gly:βCD clearly emerges as the most promising formulation. It combines the highest initial curcuminoid recovery (788.82 µgCurc/gEXT, 2-fold ChCl:Gly) with a low storage loss (−15.25%). To exemplify this, if the normalized degradation is applied to each sample’s starting yield, this corresponds to ca. 414 µgCurc/gEXT remaining after 30 days, i.e., 3-fold higher than ChCl:Gly (ca. 139 µgCurc/gEXT) and one order of magnitude above the Bet-based systems (ca. 26 and 42 µgCurc/gEXT, with and without βCD, respectively).

The enhanced stability of curcuminoids within NaDES systems is a phenomenon well-documented in the recent literature. NaDES are recognized for their capacity to stabilize labile and sensitive phytochemicals by allowing for the precise tuning of physicochemical properties (such as the utilization of acidic NaDES to preserve anthocyanins). Previous studies investigating anthocyanin extraction from complex plant matrices [11], such as violet potato, demonstrated that NaDES-based (choline chloride:lactic acid) extracts exhibited a 5.6-fold increase in shelf-life compared to ethanolic benchmarks. This preservation was rigorously evaluated through the monitoring of antioxidant capacity via DPPH· radical scavenging assays over an extended storage period. While ethanolic extracts showed a rapid decline in radical scavenging activity due to the progressive degradation of the phenolic scaffold, the NaDES media maintained high inhibition percentages throughout the months. This sustained antioxidant performance serves as a reliable proxy for the structural integrity and chemical reactivity of the native molecules; it suggests that the eutectic network effectively protects the bioactive compounds from oxidative phenomena and nucleophilic attacks that would otherwise lead to loss of function. This aligns with recent literature demonstrating that NaDES significantly enhance the stability of sensitive bioactives, such as polyphenols and flavonoids, by forming a dense hydrogen bond network that shields them from oxidative degradation [39].

In the present study, the application of a SupraDES incorporating CD into the eutectic network, further augmented the stabilizing effect beyond that of “standard” NaDES. While existing literature confirms that curcumin stability in ChCl:Gly system is significantly superior to that in alcoholic solvents, our optimized SupraDES platform provides even greater photostability and thermal resistance [40]. To compare the performance of the optimized extract, conventional organic solvent, namely EtOH, was used to benchmark the result (under the same extraction optimal conditions) which yield in higher recovery (*approx*. ten-fold). Although conventional organic solvents achieved a higher quantitative recovery, the superior long-term stability and preservation of bioactivity offered by the SupraDES platform effectively offset the lower initial extraction yield, establishing it as a more robust and sustainable alternative for bioactive delivery.

#### 2.1.4. MW-Assisted (MAE) Extraction Optimization

**Figure 5:**
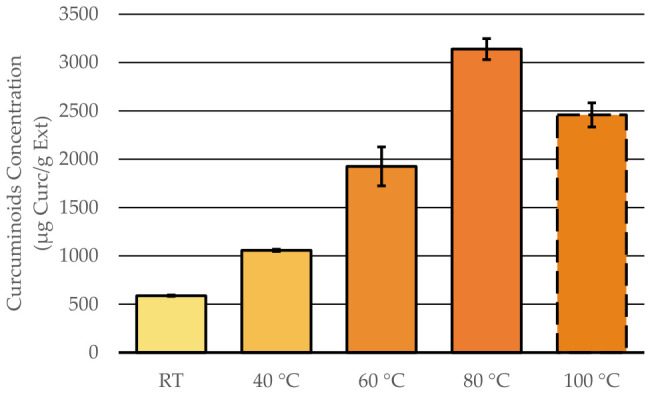
Extraction optimization, temperature screening. Fixed time: 15 min. Dashed lines indicate the degradation onset.

Given the superior performance of ChCl:Gly:βCD, the next step focused on extraction optimization, to maximize curcuminoids recovery while keeping the protocol sustainable. Firstly, a temperature MAE screening has been conducted using a fixed, short extraction time (15 min) to rapidly map the effect of temperature on extraction efficiency and identify the most promising operating window. Yields registered are reported in Figure 5.

The 15 min temperature screening shows a strong effect on curcuminoids’ recovery, with a clear optimum around 80 °C. Moving from RT to 40 °C already boosts extraction substantially, almost doubling the yield (from 587.7 to 1057.4 µgCurc/gEXT), which continue to rise at 60 °C (1926.1 µgCurc/gEXT). The maximum is reached at 80 °C (3139.4 µgCurc/gEXT), approx. 5.3-fold higher than RT (15 min). Interestingly, increasing to 100 °C triggers degradation phenomena, reducing the yield of ca. 22% in respect to the previous temperature (2458.6 µgCurc/gEXT). An additional observation supports the existence of an upper thermal stability limit for the formulation itself. To probe the intrinsic stability window of the SupraDES, considering the presence of sugar moieties in its composition, a separated blank heating/irradiation test (i.e., solvent system without curcuminoids) was performed. Notably, the sample heated at 100 °C developed a visible brownish coloration, indicating the onset of thermal caramelization. This finding is consistent with the reduced extraction yield observed at 100 °C and suggests that operating below this threshold (e.g., 80 °C) better preserves both the solvent integrity and extraction performance.

Overall, 80 °C appears the most promising temperature to carry forward into the kinetic optimization. As a final check within the DES selection, and to further substantiate the impact of βCD incorporation, a comparative extraction was performed using ChCl:Gly at 80 °C for 15 min. Under these conditions, the curcuminoid yield was 1759.4 µgCurc/gEXT, close to half of that obtained with the correspondent SupraDES, confirming the beneficial contribution of the cyclodextrin-containing system, even at mildly elevated temperature and short residence time.

**Figure 6:**
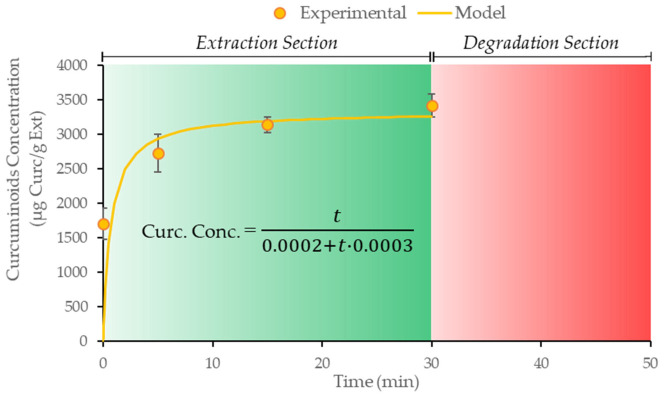
Extraction optimization, kinetic study and Peleg model extrapolation (R^2^: 0.997). Fixed temperature: 80 °C. Red area indicates the degradation onset.

Once the optimal temperature was determined, a dedicated time/kinetic study was carried out, to refine the extraction time, investigate the extraction dynamics and identify the steady-state (or near-equilibrium) under the optimized conditions. At the selected 80 °C, the kinetic profile (Figure 6) indicates a really fast initial extraction rate, followed by a clear approach to equilibrium after 10/15 min.

The curcuminoids yield increases from 1702 µgCurc/gEXT at 0 min to 2726 µgCurc/gEXT (5 min, ca.*+*60%), followed by a reducing cumulative stepwise increase (+15% and +9%, respectively), with the final output at 30 min, 3416 µgCurc/gEXT. This behaviour is consistent with a rapid solubilization step followed by a slower diffusion-controlled stage, suggesting that 15–30 min is the practical operating window at 80 °C, 15 min as an efficiency-driven compromise, considered that doubling the time do not lead to a suitable yield increase. After this considered time range, 60 min led to curcuminoids’ degradation, and complementary blank tests confirm that prolonged thermal stress affects the SupraDES system itself: ChCl:Gly:βCD at 80 °C for 60 min becomes yellowish, whereas the 15 min blank remains unchanged. The control experiment (ChCl:Gly at 80 °C for 60 min) remained transparent, indicating that the alteration its related to βCD, consistent with the previously observed browning at 100 °C and supporting a thermal stability ceiling for βCD-containing formulations, both in the temperature and in the time-frame.

To go beyond a purely empirical comparison of yields, we modelled the kinetic dataset to extract physically meaningful descriptors of the extraction process (e.g., extraction rate, k_1_ and extraction capacity, ∝k_2_). For that task, Peleg model has been chosen because it is one of the most widely adopted, a robust semi-empirical approach for fitting extraction kinetics in the literature, enabling straightforward benchmarking against previous studies [29,41,42].

The Peleg model parameters (k_1_ = 0.0002; k_2_ = 0.0003) further corroborate this interpretation: the low k_1_ reflects a high initial extraction rate, while k_2_ implies a theoric asymptotic capacity (1/k_2_) of *approx.* 3333 µgCurc/gEXT, aligning well with the observed near-plateau between 15 and 30 min. Overall, the kinetics and blank stability tests jointly justify selecting 80 °C with short residence times to maximize recovery while minimizing both curcuminoid degradation and SupraDES thermal alteration.

### 2.2. PVA-Based Biopolymers

The environmental challenges associated with the widespread use of petrol-based non-biodegradable plastics, raised interests toward bio-based materials for food (active) packaging. Among all the available alternatives, PVA represents a promising option due to its biodegradability, non-toxicity, and excellent physical, mechanical, and film-forming properties. PVA is often blended with other bio-derived components, such as chitosan, gelatin, and cellulose, in order to further enhance the sustainability and functionality of the resulting materials [43].

To improve the mechanical features of these polymers, Gly is commonly employed as a plasticizer, while maintaining biodegradability and compliance with food contact regulations [44,45]. Another additive commonly used in combination with PVA to enhance mechanical behaviour of the polymer, including gelling, thickening, and stabilizing effect, is pectin. Several studies reported in the literature have explored the fabrication of PVA-pectin-based polymers for food packaging applications. Among them Suhasini et al. incorporated MgO together with pectin to improve film texture, mechanical strength and thermal stability of films [46]. Similarly, Teleki et al. investigated the development of active packaging materials enriched with itaconic acid (as crosslinking agent) and apple pomace-derived antioxidants, to obtain a biodegradable and water-soluble packaging films [47]. An active material composed of PVA and cyclodextrin was developed as a packaging system capable of reducing the content of undesirable compounds, such as cholesterol, in foods through the active retention of these molecules within the packaging matrix during storage [48]. Beyond these specific applications, cyclodextrins are commonly included in polymeric material, alongside bioactive molecules to improve their solubility, to guarantee the homogeneous distribution of the complexed molecules. Furthermore, the formation of cyclodextrin inclusion complexes protects these compounds from volatilization, oxidation, and temperature fluctuations when incorporated into polymeric systems [49].

Another component of the prepared biopolymers is BHMF, a bio-derived crosslinker, obtained by reduction of 5-hydroxymethilfurfural (HMF) [50]. This bio-based platform chemical derived from renewable sources such as sugars, have gained increasing attention in last years. Several studies have investigated different polymerization strategies for BHMF-based polyesters, evaluating their biodegradability and toxicity [51]. BHMF has also been combined with other polymeric building blocks such as PVC, where it can act as a plasticizer [52]. Coming to the main constituent of the polymer, the bulk framework is represented by a ChCl-based SupraDES, according to already existing literature. In particular ChCl:Gly have already been investigated for the formation of a bioactive polymer, alongside ChCl:CitA, demonstrating good biodegradability and low toxicity [53,54]. These results support the potential application of the polymers described here in food packaging, while also enabling the exploitation and stabilization of curcuminoid bioactivity.

#### 2.2.1. Biopolymer Fabrication

Building on the identification of ChCl:Gly:βCD as the most effective medium for both curcuminoid extraction and stabilization, the study advances the second facet of the Janus solvent concept: a “solvent-to-material” strategy, in which the same eutectic platform is valorized as a functional component in biopolymer fabrication. Beyond the established role of DES as extraction media, recent studies describe selected ChCl-based NaDES as enabling systems for polymer synthesis, acting not only as solvents but also as reactive media that yield βCD-based polymers subsequently cured into crosslinked networks [30]. In this framework, the curcuminoid-loaded ChCl:Gly:βCD SupraDES is investigated as a multifunctional ingredient, combining a carrier/protective microenvironment (host–guest and eutectic solvation) with a supramolecular structuring/plasticizing phase, thereby translating extraction performance into a stable, application-oriented material. Notably, the cited NaDES-to-polymer literature mainly relies on ChCl:CitA formulations, where CitA functions as a reactive crosslinking unit via anhydride intermediates, often promoted by catalysts (i.e., sodium hypophosphite). A formulation screening was therefore carried out, leveraging prior experience on PVA-based scaffolds combined with bio-based additives (e.g., pectin and tannins) [55], with the key challenge of obtaining a robust polymeric framework while mitigating the high glycerol content inherited from the base eutectic mixture. For this reason, although CitA is not considered favourable for curcuminoid stability, it was introduced during the polymerization stage, exploiting its well-established ability to crosslink polyols/polysaccharides/PVA through ester bonds upon thermal curing (i.e., CitA–PVA [56], CitA–pectin [57], CitA–Gly [58]). Among the investigated components, the bio-derivable reactive diol BHMF was selected to further enhance the sustainability profile of the final material [53]. To further improve the sustainability of the polymer fabrication process, the pectin fraction was recovered from orange peel waste by subcritical water extraction, according to the protocol described in a previous study [55].

Different combinations of the above components were tested as a preliminary step to identify compositions achieving adequate biopolymer consistency; the outcomes and relative amounts are reported in Table 3.

The screening highlights a clear binary gate for obtaining a stable solid: CitA and βCD must be present. In both extracts (SupraDES and CD-free NaDES), all formulations lacking CD (Samples 1–3 and 5–7) remained liquid, even when CitA and pectins were included, confirming that βCD provides a decisive contribution to network build-up beyond the eutectic phase alone. Conversely, CD + CitA enabled solid formation (Samples 4 and 8), as well as in the corresponding controls (4_Control, 8_Control).

Within this screening, BHMF was not required for “formation”, since both controls yielded stable solids without it, suggesting that its role is better framed as a sustainability/chemistry modifier (reactive diol) rather than a prerequisite for gelation/crosslinking. Thus, BHMF does not provide alone enough multifunctional hydroxyl density and/or network connectivity to build a self-standing polymeric framework on its own.

Notably, all successful formulations contained pectin, suggesting its role as a polysaccharide backbone within the polymer network. This structure likely cooperates with CitA-driven ester crosslinking involving PVA and pectin hydroxyl groups, while β-CD may contribute to supramolecular organization of the network. Consequently, the DES extract is converted into a robust polymeric framework. CitA-based DES systems are known to act as reactive media for βCD-containing polymer networks, particularly in ChCl:CitA formulations, where curing promotes ester-type crosslinking [30]. However, comparable evidence for betaine-based analogues (Bet:CitA), either in presence or not of βCD, appears limited or absent, despite betaine being an attractive bio-derived HBA. For this reason, a focused side-screening was performed to verify whether Bet:CitA systems can also act as robust polymer-forming platforms in the condensation conditions here exploited (Table 4). It worth noting that these tests are independent from the unsuitable performance of Bet-based DES in curcuminoid extraction. Pectins were additionally included in selected recipes as an extra –OH-rich biopolymeric source, consistent with the previous formulation logic.

All formulations in Table 4 led to successful formation of stable solids (Samples 9–12), both in the NaDES and SupraDES variants. This indicates that, in this chemical space, polymer formation is readily enabled by the Bet:CitA reactive environment and, differently from the Gly-based systems, βCD is not a prerequisite to reach sufficient condensation. The inclusion of pectin did not alter the formation of the biopolymer, but resulted in opaque materials (Samples 10 and 12), plausibly due to increased network heterogeneity and light scattering arising from polysaccharide-rich domains. Overall, these results support the view that CitA-driven crosslinking chemistry dominates the formation behaviour in Bet:CitA systems. In addition, the PVA/CitA ratio in these formulations is likely sufficient to generate efficient interaction, and this effect may be further favoured by the absence of Gly, providing a reduced overall polyol concentration, allowing PVA (and pectins, when present) to contribute more directly to network build-up during curing.

#### 2.2.2. Biopolymer Characterization

**Figure 7:**
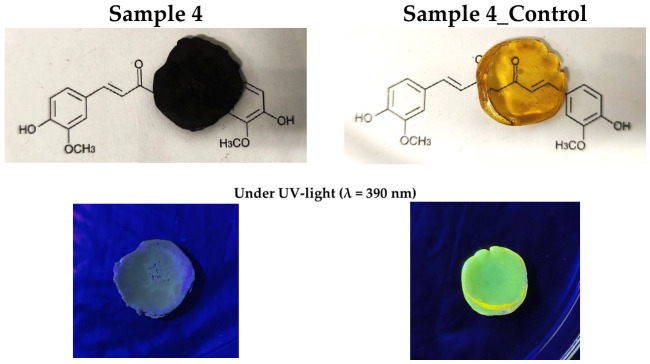
Fabricated polymers Sample 4 and Sample 4_Control. UV irradiation (390 nm) and related fluorescence of the supported curcuminoids.

The visual characteristics of the two formulations, 4 and 4_control (with and without BHMF), are shown in the following figures (Figure 7). As observed, 4_control is a highly transparent material, whereas the incorporation of the diol resulted in a distinct colour shift toward dark brown. Both polymers were subjected to UV irradiation (λ = 390 nm)) to qualitatively evaluate the fluorescence of the integrated curcuminoids. While 4_control exhibited intense fluorescence, the signal from sample 4 was attenuated by the darker framework, though emission remained detectable. This optical responsiveness suggests that the curcuminoids can function as an integrated fluorescent sensor system, for the possible development of smart and active packaging capable of monitoring material integrity [59].

**Figure 8:**
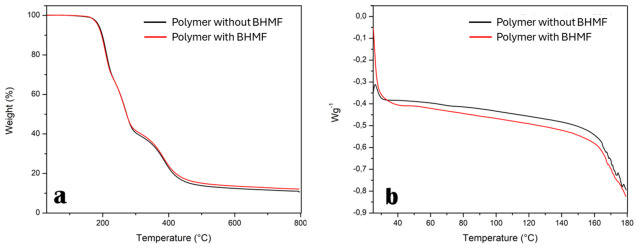
Thermal analysis of the polymeric systems with and without BHMF. (**a**) TGA curves of the polymer without BHMF (black line) and the polymer containing BHMF (red line). (**b**) DSC thermograms of the polymer without BHMF (black line) and the polymer containing BHMF (red line).

Thermal analysis was performed to characterize the polymer network in the absence and in the presence of BHMF. The TGA and DSC results are reported in Figure 8.

The TGA curves of the two materials, namely the polymer without BHMF and the polymer containing BHMF, exhibit very similar thermal profiles, indicating that the incorporation of BHMF does not significantly affect the thermal stability of the polymer network. In both samples, the onset of degradation occurs at approximately 180 °C.

This temperature can be associated with the cleavage of ester bonds formed during the crosslinking reaction between citric acid and the hydroxyl groups present in PVA, cyclodextrin, and, in the modified system, BHMF. The abundance of hydroxyl functionalities in these components promotes esterification during the synthesis, leading to the formation of a crosslinked network. Therefore, the initial mass loss observed around 180 °C is likely related to the thermal scission of these ester linkages. At higher temperatures, a second degradation step is observed at around 300 °C.

This weight loss can be attributed to the progressive decomposition of the polymeric matrix, including the degradation of the PVA backbone and of the carbohydrate-based components (citric acid and cyclodextrin) forming the network. The similarity of the degradation profiles of the two systems further confirms that the introduction of BHMF does not substantially modify the overall stability of the polymer.

Regarding the DSC analysis, the two samples exhibit very similar thermal profiles, confirming that the incorporation of BHMF does not significantly alter the thermal behaviour of the polymeric network. No significant thermal transitions are observed in the temperature range between 150 and 180 °C. This absence of detectable peaks suggests that no additional reactions occur within this temperature interval. This behaviour can be attributed to the absence of free hydroxyl groups available for further reactions. During the synthesis process, the hydroxyl functionalities present in PVA, cyclodextrin, and BHMF react with the carboxylic groups of citric acid to form ester linkages, resulting in the formation of a crosslinked polymer network. As a consequence, the esterification reaction is likely completed during the synthesis stage, and no further polymerization or crosslinking processes occur upon heating in the DSC experiment. Therefore, the DSC results further support the formation of a stable crosslinked structure and are consistent with the TGA data, indicating that the presence of BHMF does not significantly influence the overall thermal behaviour of the polymer.

**Figure 9:**
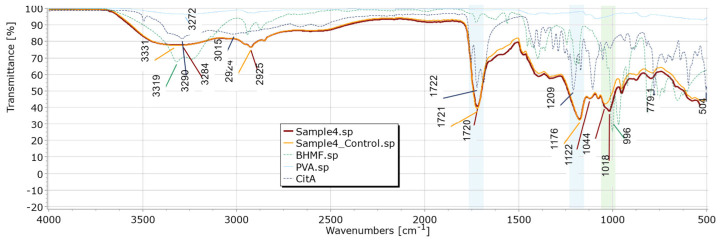
ATR-FTIR analysis of the polymers: Sample4 (brown line), Sample4_Control (yellow), BHMF (dotted green), PVA (light blue) and citric acid (dotted dark blue). Light blue area points out the diagnostic peaks for esters while light green area points out the diagnostic peaks for BHMF.

Sample4 and Sample4_control have been characterized also by means of AFT-FTIR investigation (see Figure 9).

Sample4 and Sample4_Control were characterized *via* FTIR, together with three of the components of the polymer, namely PVA, CitA and BHMF.

The OH stretching region, ranging from 3100 to 3300 cm^−1^ is notably crowded; however, several key observations can be made. The sharp peaks originally associated with BHMF and CitA were smoothed during the reaction. The resulting polymer profiles exhibit a broader, more continuous peak in this region, closely resembling that of PVA, yet shifted relative to the base polymer. This shift confirms that a reaction occurred between the hydroxyl groups of the PVA and the other compounds.

Regarding the formation of the polymer network, the literature identifies the signal at 1720–1721 cm^−1^ as clearly attributable to the ester formation and terminal carboxyl group [30]. This confirms a distinct change between the original spectrum of CitA, in the same region. Accordingly, the signal present at 1122 cm^−1^ is assigned to C-O-C stretching vibration of ester groups [60], while the peak at 1176 cm^−1^ suggests the presence of free carboxyl functions, from the free terminals of CitA molecules [61].

These results verify the formation of the polymer backbone, primarily driven by the esterification of CitA. These observations apply to both Sample 4 and Sample 4-control; as shown in the spectra above, the profiles largely overlap, particularly in the aforementioned regions. The signal at 1044 cm^−1^ is a characteristic peak attributable to CH_2_-O, which is largely present in both samples due to the backbone of PVA [62]. However, in the Sample 4 (containing BHMF) the signal is deformed by the adjacent shoulder of furan signals, which can be attributable to the furan ring breathing signal (symmetric stretching of the furan ring itself), at ca. 1018 cm^−1^ [60] or to the CH_2_ wagging of 2,5-disubstituted furans [63].

## 3. Materials and Methods

### 3.1. Materials and Reagents

*Curcuma longa* L. powder was purchased as commercially available feedstock. The granulometry of the powder, obtained after hammer mill treatment, was below 2 mm. Before use, the biomass was stored at RT and in darkness, and all handling procedures were performed under dark conditions. All the reagents were purchased by Sigma-Aldrich: choline chloride (ChCl), purity ≥ 98%, freeze-dried before use; betaine (Bet); glycerol (Gly), purity ≥ 99.5%; citric acid (Cit), purity ≥ 99.5%; β-Cyclodextrin, purity ≥ 97%; curcumin standard grade. Absolute ethanol was purchased from VWR GmbH (Darmstadt, Germany).

### 3.2. Preparation of Natural Deep Eutectic Solvents (NaDES) and Supramolecular Eutectic Solvents (SupraDES)

The prepared solvents hereafter described, can be classified as NaDES, as they are composed of naturally occurring or bio-derived components, including ChCl, Gly, Bet, CitA, and βCD. In particular, when the solvent is prepared in combination with cyclodextrins (ternary component), it is referred to as SupraDES, according to the literature [16]. For the sake of simplicity NaDES is normally used for the ones prepared without βCD. When the text makes no distinction between NaDES/SupraDES, the common definition adopted here is DES.

DESs were prepared using a microwave (MW)-assisted multimode reactor (SynthWAVE, Milestone, Bergamo, Italy). The system allows a controlled environment, ensuring constant temperature, agitation and rotation, to rapidly prepare the eutectic solution in the most efficient way, in an inert atmosphere (under nitrogen backpressure). The various components that were selected as HBA and HBD, were accurately weighed and inserted in a glass vial. Various DES were prepared using different combinations of HBA e and HBD, in particular ChCl:Gly, ChCl:Cit, Bet:Gly and Bet:Cit, along with the same formulations within addition β-cyclodextrin. The molar ratios of each component are reported in Section 2.1.1. Components were preventively freeze-dried (Lyotest −85 no plus, Telstar, Spain) to eliminate any excess water that may have been present, due to hygroscopicity, to guarantee a controlled composition of water in the DES.

The operative conditions concerning temperature and time were optimized according to the various compositions of each DES but, indicatively, all were firstly heated at 60 °C for 15 min. If this was sufficient to obtain a clear DES, the sample was removed; otherwise, subsequent treatments at higher temperatures (60 °C → 80 °C → 100 °C) were applied. The specific conditions for each DES are reported in Table 5.

At the end of the process, the DES were stored in closed glass vials and preserved from humidity, at RT until use.

### 3.3. NaDES/SupraDES-Assisted Curcuminoid Extraction

#### 3.3.1. Component Selection

Curcuminoid extraction was carried out typically on 100 mg of *Curcuma Longa* L. powder, mixed with 2 g of DES (S/L 1:20). Each mixture was subjected to constant stirring at RT for 2 h in dark conditions. The obtained suspensions were previously centrifuged (Eppendorf tubes, 4200 rpm, 5 min, Cence Hunan Xiangyi Laboratory Instrument Development Co., Ltd., Changsha, China). The supernatant was filtered using a cotton-packed syringe (VWR GmbH, Darmstadt, Germany) to remove any residual particles. The obtained clarified extracts were then transferred into closed, amber-glass vials to prevent degradation and kept at RT for further analyses.

#### 3.3.2. MW-Assisted (MAE) Extraction Optimization

The selected DES underwent MAE optimization, exploiting SynthWAVE reactor to fasten the screening and to preserve the quality of the extract, working under N_2_ atmosphere (2 bar). The treatment time was fixed at 15 min, and the extraction at RT was repeated, while different temperatures (40, 60, 80, and 100 °C) were also investigated. After the extraction, samples underwent the same downstream process as previously described in Section 3.3.1. All the extractions were performed in triplicate and the results expressed as average ± the standard deviation.

#### 3.3.3. Kinetic Studies

**Figure A1:**
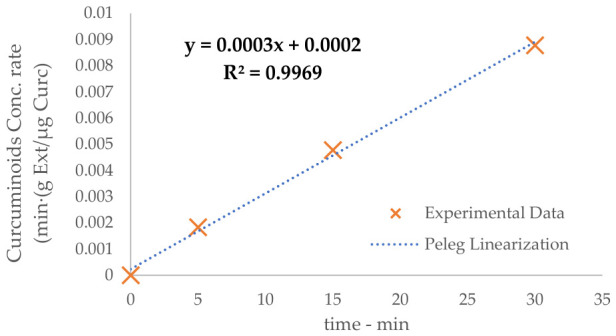
Peleg linearization and fitting information.

**Figure A2:**
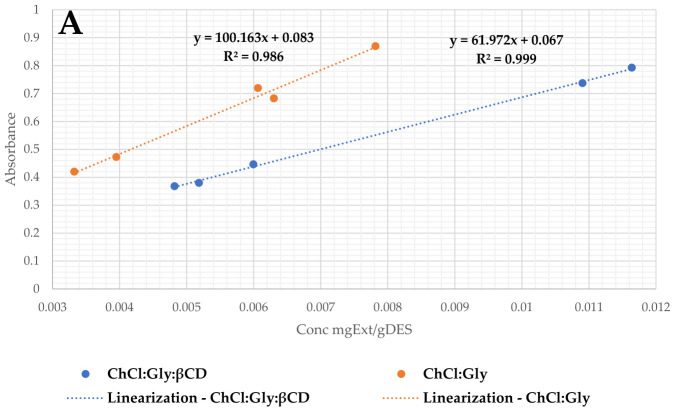

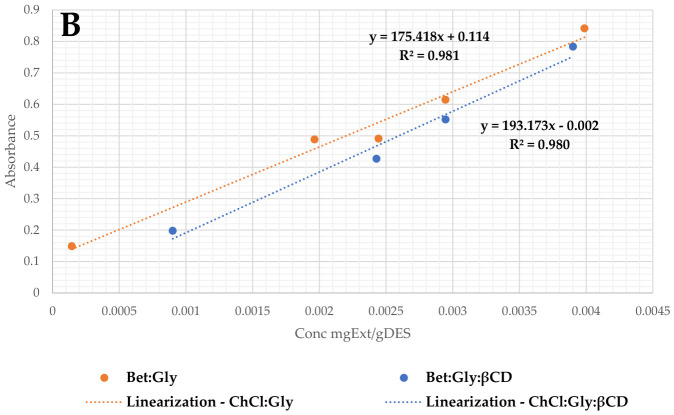
Extinction coefficient (ε) extrapolation. (**A**): ChCl:Gly vs. ChCl:Gly:βCD; (**B**): Bet:Gly vs. Bet:Gly:βCD.

Once the optimal temperature was selected, a kinetic study was performed to describe the time effect on the procedure, assessing extraction rate and equilibrium set point as the highest achievable yield. The investigation involved time 0 min, 5, 15, 30 and 60 min. All the extractions and the downstream processes were performed as previously described. All the extractions were performed in triplicate and the results expressed as average ± the standard deviation. To assess the kinetic model, experimental data were fitted with Peleg’s model by linearization, extrapolating the two kinetic constants: (i) k_1_ as the extraction rate and (ii) k_2_ as inversely proportional to the theoretic asymptotic extraction capacity [64]. Linearization and fitting information are reported in Appendix A (Figure A1).

### 3.4. UV-Vis Spectrophotometric Analysis

#### 3.4.1. Calibration Curve of Curcumin in DES

For the preparation of the calibration curve, a standard solution was prepared by dissolving 0.5 mg of standard curcumin in 1 g of each DES. Subsequently, from this stock solution a dilution series was prepared, to create a 4-point calibration curve. All spectrophotometric measurements were performed using UV-Vis (Cary, Agilent, Santa Clara, CA, USA), with absorbance recorded at a diagnostic wavelength of 425 nm, and using pure DES as blank [65]. Quantification was carried out using curcumin as a reference standard, and results are expressed as curcumin equivalents, in line with established analytical practices for structurally related compounds sharing a common chromophore [65]. Before the measurement, the cuvettes containing the diluted solutions were centrifuged for 5 min to eliminate bubbles that could interfere with the measurement. The linearity of each curve was validated through the R^2^ value (≥0.98) and the absorbance coefficient (*ε*) was calculated in Equation (1):

(1) $$\varepsilon =\frac{m}{l}$$

where *m* is the slope of the curve and *l* is the optical path length (1 cm). Since the concentrations used to build the curve were expressed as μgCurc/gDES, ε does not represent the molar absorptivity coefficient but is expressed accordingly (μgCurc/gDES).

The obtained coefficient was subsequently used, applying the Lambert–Beer law, to determine the concentrations from the measured absorbance values, using Equation (2):

(2) $$C=\frac{A}{\varepsilon \cdot l}$$

Thus, the concentration (*C*) is expressed as a weight-on-weight ratio, between curcumin equivalents and DES (generally expressed as a selectivity, μgCurc/gEXT).

#### 3.4.2. Quantification of Curcuminoids in DES Extracts

The curcuminoid extracts were analyzed by adopting the same procedure and carrying out dilutions to acquire readings in the linearity range. Before the measurement, the cuvettes containing the diluted extracts were centrifuged for 5 min to eliminate bubbles that could interfere with the measurement. For each DES at least 3 points were obtained to create a curve, and the resulting linearity was used to evaluate the reliability of the measurement. To extrapolate the concentration of each sample, related ε was used.

### 3.5. Shelf-Life

The stability of the curcuminoids contained in the extracts was evaluated under standard storage conditions (RT). Stability was assessed for all eight DES-based extracts after 30 days of storage in amber-glass vials. After the incubation period the UV-Vis was used to extrapolate the percentage of degradation, with respect to the initial concentration (results express as % of degradation).

### 3.6. PVA-Based Biopolymers

#### 3.6.1. Component Formulation

To prepare the PVA-based biopolymer, several combinations of components were tested (see Table 6 and Table 7), including two family of DESs: (i) the best-performing one and its reference without βCD (ChCl:Gly:βCD and ChCl:Gly, respectively), and (ii) Bet:Cit-based DESs, as a preliminary investigation for future studies. Among the investigated components, the bio-derivable BHMF was selected to evaluate its applicability.

The amount of CitA added to the polymer formulation was adjusted to match the adopted ChCl:CitA literature molar ratio.

#### 3.6.2. Biopolymer Fabrication

**Figure 10:**
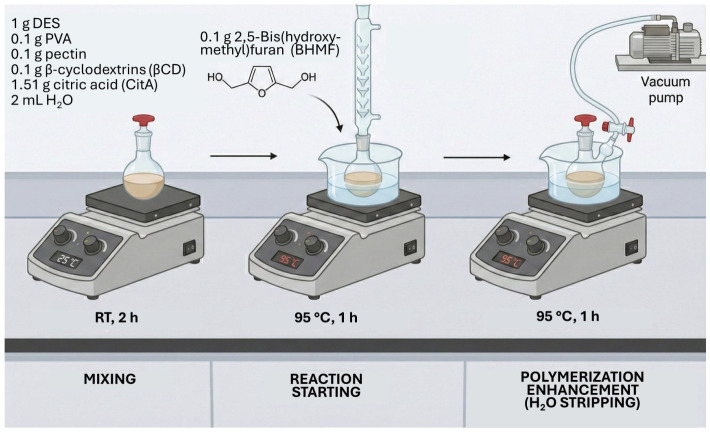
Schematic representation of the biopolymer fabrication protocol.

A total of 1 g of the optimized SupraDES extract (ChCl:Gly:βCD), together with the selected components, namely 0.1 g of PVA, 0.1 g of pectin, 1.51 g of CitA (7.86 mmol), 0.1 g of βCD (0.088 mmol) and 2 mL of water, were added to a round-bottom flask and stirred at RT for 1 h. At the end of the mixing period, 0.1 g of BHMF (0.78 mmol) was added. The mixture was then placed in a silicone oil bath on a hot plate, and the flask was equipped with a Vigreux condenser. The mixture was heated to 95 °C and magnetically stirred for 1 h. Subsequently, the mixture was kept under vacuum for 1 h to remove the water formed by the condensation reaction and shift the equilibrium toward the products. Finally, the viscous prepolymer was cast and left to cure for 48 h at 45 °C and for 12 h at 80 °C. The procedure is schematically illustrated in Figure 10.

#### 3.6.3. Biopolymer Characterization

The biopolymer was characterized by means of thermal analysis in order to evaluate its thermal stability, phase transitions and curing. In particular, samples of the neat polymer and polymer containing BHMF were analyzed by thermogravimetric analysis (TGA) and differential scanning calorimetry (DSC). TGA measurements were performed using a TGA Q500 instrument (TA Instruments, New Castle, DE, USA). Approximately 10 mg of each sample were placed in aluminum pans and heated from 30 to 800 °C at a heating rate of 10 °C min^−1^ under a nitrogen atmosphere (flow rate: 100 mL min^−1^) to prevent oxidative degradation.

DSC analyses were carried out using a DSC Stare System (Mettler Toledo, Milan, Italy). Samples (approximately 5 mg) were sealed in aluminum pans and subjected to heating from 30 to 180 °C at a rate of 10 °C min^−1^ under a nitrogen atmosphere (flow rate: 50 mL min^−1^).

ATR-FTIR spectra were obtained using ATR equipped with a Smart Endurance diamond crystal (Spectrum Two ATR, Perkin Elmer, Waltham, MA, USA). Data were collected from 500 to 4000 cm^−1^ over 24 scans in transmittance mode.

## 4. Conclusions

To the best of our knowledge, this proof-of-concept study is the first report that βCD-based Supramolecular Deep Eutectic Solvents (SupraDES) can serve as a Janus green platform, integrating curcuminoid extraction with subsequent biopolymer fabrication within a unified solvent–material strategy, without preliminary purifications.

According to the observed trends, ChCl:Gly:βCD delivered the best combined performance among all tested solvents, emerging as the clear lead formulation. It provided the highest extraction efficiency already at RT (788.82 µgCurc/gEXT, more than 2-fold ChCl:Gly and far above the betaine-based systems) while also ensuring superior shelf-life, with markedly lower 30-day degradation (47.52% vs. 62.77% for ChCl:Gly). This SupraDES was therefore selected for further optimization. Temperature screening identified 80 °C as the most promising operating point, and direct benchmarking at 80 °C/15 min confirmed that βCD incorporation remains beneficial under intensified conditions, with ChCl:Gly:βCD consistently outperforming the βCD-free NaDES.

On the material side of the Janus platform, polymer screening showed that for Gly-based extracts, CitA addition and βCD act as key components, whereas BHMF is not sufficient as a polyol source and does not lead to solidification. Conversely, Bet:CitA systems readily formed condensed biopolymers even without βCD, consistent with CitA-driven crosslinking and effective PVA interaction in the absence of Gly excess. As confirmed by thermal analysis, TGA and DSC, a stable crosslinked structure was generated. The esterification reaction between components is likely completed during the synthesis step, both for Sample 4 and 4_control, indicating that the presence of BHMF has a negligible impact on the polymer thermal behaviour. Further studies may be needed in the future to better investigate structure–properties relations. The collected evidence, together with the nature of the biopolymer’s components, could pave the way to further studies, devoted to investigate their applicability in different fields, such as active packaging or food-related uses.

## Figures and Tables

**Table 1:** NaDES/SupraDES composition and relative stability.

Acronym	HBA	HBD	βCD	Mol Ratio *	Formation ^§^
					(°C)
ChCl:Gly	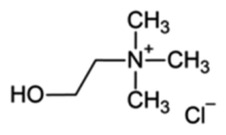	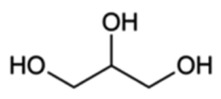	N	1:1	80
ChCl:Gly:βCD			Y	1:1:0.02	60
Bet:Gly	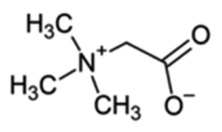	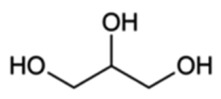	N	1:2	60
Bet:Gly:βCD			Y	1:2:0.02	60
ChCl:CitA	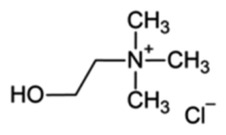	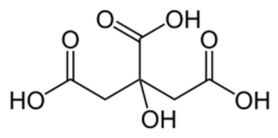	N	1:2	100
ChCl:CitA:βCD			Y	1:2:0.02	80
Bet:CitA	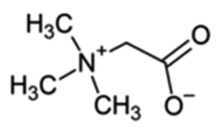	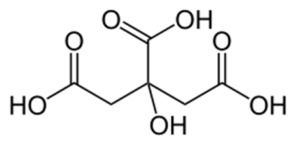	N	1:2	100
Bet:CitA:βCD			Y	1:2:0.02	80

* all DES contain 10% H_2_O *w/w*; ^§^ heating time: 15 min.

**Table 2:** Gly-based NaDES/SupraDES *ε* extrapolation at 425 nm.

NaDES/SupraDES	ε@425 nm (µgCurc/gDES·cm)^−1^	R^2^
ChCl:Gly	100.16	0.986
ChCl:Gly:βCD	61.97	0.999
Bet:Gly	175.42	0.981
Bet:Gly:βCD	193.17	0.980

**Table 3:** Fabrication of extract’s biopolymer, based on ChCl:Gly:βCD (SupraDES) and related NaDES reference (ChCl:Gly).

Core Composition	Sample	CitA	Pectin	BHMF	βCD	Formation
SupraDES extract (ChCl:Gly:βCD + PVA + H_2_O)	1	Y	N	Y	N	X
	2	N	Y	Y	N	X
	3	Y	Y	Y	N	X
	4	Y	Y	Y	Y	✓
	4_Control	Y	Y	N	Y	✓
NaDES extract (ChCl:Gly + PVA + H_2_O)	5	Y	N	Y	N	X
	6	N	Y	Y	N	X
	7	Y	Y	Y	N	X
	8	Y	Y	Y	Y	✓
	8_Control	Y	Y	N	Y	✓

**Table 4:** Fabrication of biopolymer based on Bet:Gly:*β*CD (SupraDES) and related NaDES reference (Bet:Gly).

Core Composition	Sample	Pectin	Formation
SupraDES (Bet:CitA:βCD + PVA + H_2_O + BHMF)	9	N	✓
	10	Y	✓ (opaque)
NaDES (Bet:CitA + PVA + H_2_O + BHMF)	11	N	✓
	12	Y	✓ (opaque)

**Table 5:** Preparation of DES: sequential treatments necessary to prepare the DES until formation of clear solution.

DES	Sequential Treatments (15 min) *		
	60 °C	80 °C	100 °C
ChCl:Gly	X	✓	-
ChCl:Gly:βCD	✓	-	-
ChCl:CitA	X	X	✓
ChCl:CitA:βCD	X	✓	-
Bet:Gly	✓	-	-
Bet:Gly:βCD	✓	-	-
Bet:CitA	X	X	✓
Bet:CitA:βCD	X	✓	-

* Sequential treatments applied during DES formation. “X” indicates unsuccessful formation under the applied conditions, requiring a subsequent treatment at higher temperature until DES formation (✓).

**Table 6:** Fabrication of extract’s biopolymer, based on ChCl:Gly:βCD (SupraDES) and related NaDES reference (ChCl:Gly): components and quantities.

Base	Sample	DES	PVA	H_2_O	CitA	Pectin	BHMF	βCD
SupraDES (ChCl:Gly:βCD)	1	1 g	0.1 g	2 mL	1.51 g	-	0.1 g	-
	2				-	0.1 g	0.1 g	-
	3				1.51 g	0.1 g	0.1 g	-
	4				1.51 g	0.1 g	0.1 g	0.1 g
	4_Control				1.51 g	0.1 g	-	0.1 g
NaDES (ChCl:Gly)	5	1 g	0.1 g	2 mL	1.51 g	-	0.1 g	-
	6				-	0.1 g	0.1 g	-
	7				1.51 g	0.1 g	0.1 g	-
	8				1.51 g	0.1 g	0.1 g	0.1 g
	8_Control				1.51 g	0.1 g	-	0.1 g

**Table 7:** Fabrication of biopolymer based on Bet:Gly:βCD (SupraDES) and related NaDES reference (Bet:Gly): components and quantities.

Base	Sample	DES	PVA	H_2_O	BHMF	Pectin
SupraDES (Bet:CitA:βCD)	9	1 g	0.1 g	2 mL	0.1 g	-
	10					0.1 g
NaDES (Bet:CitA)	11	1 g	0.1 g	2 mL	0.1 g	-
	12					0.1 g

## Data Availability

Data are contained within the main manuscript and in Appendix A.

## Abbreviations

The following abbreviations are used in this manuscript:

Bet	Betaine
βCD	β-cyclodextrin
ChCl	Choline Chloride
CitA	Citric acid
DES	Deep Eutectic Solvents
BHMF	2,5-bis(hydroxymethyl)furan
Gly	Glycerol
HBA	Hydrogen bond acceptors
HBD	Hydrogen bond donors
MAE	Microwave-assisted extraction
NaDES	Natural Deep Eutectic Solvents
PVA	Polyvinyl alcohol
SupraDES	Supramolecolar Deep Eutectic Solvents
**ε**	Extinction coefficient
